# Rating the Quality of Smartphone Apps Related to Shoulder Pain: Systematic Search and Evaluation Using the Mobile App Rating Scale

**DOI:** 10.2196/34339

**Published:** 2022-05-26

**Authors:** Jonathon M R Agnew, Chris Nugent, Catherine E Hanratty, Elizabeth Martin, Daniel P Kerr, Joseph G McVeigh

**Affiliations:** 1 Discipline in Physiotherapy School of Life and Health Sciences University of Ulster Newtownabbey United Kingdom; 2 Discipline in Computing School of Computing University of Ulster Newtownabbey United Kingdom; 3 Discipline in Physiotherapy School of Clinical Therapies, College of Medicine and Health University College Cork Cork Ireland

**Keywords:** mobile app, shoulder pain, mHealth, Mobile App Rating Scale, mobile phone

## Abstract

**Background:**

The successful rehabilitation of musculoskeletal pain requires more than medical input alone. Conservative treatment, including physiotherapy and exercise therapy, can be an effective way of decreasing pain associated with musculoskeletal pain. However, face-to-face appointments are currently not feasible. New mobile technologies, such as mobile health technologies in the form of an app for smartphones, can be a solution to this problem. In many cases, these apps are not backed by scientific literature. Therefore, it is important that they are reviewed and quality assessed.

**Objective:**

The aim is to evaluate and measure the quality of apps related to shoulder pain by using the Mobile App Rating Scale.

**Methods:**

This study included 25 free and paid apps—8 from the Apple Store and 17 from the Google Play Store. A total of 5 reviewers were involved in the evaluation process. A descriptive analysis of the Mobile App Rating Scale results provided a general overview of the quality of the apps.

**Results:**

Overall, app quality was generally low, with an average star rating of 1.97 out of 5. The best scores were in the “Functionality” and “Aesthetics” sections, and apps were scored poorer in the “Engagement” and “Information” sections. The apps were also rated poorly in the “Subjective Quality” section.

**Conclusions:**

In general, the apps were well built technically and were aesthetically pleasing. However, the apps failed to provide quality information to users, which resulted in a lack of engagement. Most of the apps were not backed by scientific literature (24/25, 96%), and those that contained scientific references were vastly out-of-date. Future apps would need to address these concerns while taking simple measures to ensure quality control.

## Introduction

Shoulder pain is one of the most common musculoskeletal complaints [1], with prevalence rates ranging from 1% to 67% among varying populations [2]. As the shoulder helps to stabilize the upper arm for many activities, ongoing shoulder pain can have a significant negative effect on daily activities, such as getting dressed, and can potentially result in poor psychological factors, such as anxiety around pain [3]. These altered beliefs around pain can lead to poorer treatment outcomes in the long term, increasing the risk of shoulder pain becoming chronic [4]. Surgical input (eg, decompression surgery) and the use of nonsteroidal anti-inflammatory drugs were previously seen as the gold standard of shoulder pain management [5]. However, there is evidence suggesting that the use of conservative treatments, such as exercise therapy and musculoskeletal physiotherapy, can be as effective as surgery and has fewer associated risks, such as the risk of infection [6]. These conservative interventions can include measures such as exercise therapy and physiotherapy techniques, including electrotherapy (eg, ultrasound), and manual therapies such as spinal and peripheral joint mobilizations, soft tissue release, muscle energy techniques, and taping [7]. However, obtaining sufficient levels of support via face-to-face appointments is not feasible due to a lack of time, demand constraints, and the recent COVID-19 global pandemic [8]. Therefore, the importance of prescribing exercise therapy for shoulder pain has increased to provide better outcomes for patients [9]. However, the evidence for the benefits of exercise for shoulder pain is still inconclusive [10]. To achieve the best long-term outcomes, it is important to encourage self-management, as this is an important predictor of successful rehabilitation [11].

Technology-based interventions for pain management, such as mobile health interventions, are an effective way of providing such self-management skills and education to both patients and health care providers [12,13]. This branch of health care is becoming increasingly popular due to the high availability of smartphone devices [14]. Mobile health has helped to increase the accessibility and affordability of health care for those living in rural locations or on low incomes [15]. This is especially important due to the previously mentioned COVID-19 pandemic, as many patients still require ongoing treatment despite the lack of face-to-face appointment availability [16]. Clear guidelines are given to app developers via The Developer Program Policies, alongside the Developer Distribution Agreement [17], to ensure the inclusion of appropriate content and the fair use of users’ data. However, the poor enforcement of these guidelines leads to the market saturation of poor-quality apps with no scientific backing [18]. As a result, despite the availability of thousands of commercially available apps related to pain management, there is no published guidance for health care professionals on how to identify a user-friendly, evidence-based app for patients [19].

The aim of this work is to provide an overview of apps related to shoulder pain that have been reviewed by using the Mobile App Rating Scale (MARS) [20]. The focus of this study is to provide new information on the quality of the content and aesthetics of currently available apps related to shoulder pain and to identify the most engaging and appealing aspects of these apps. Our findings will help guide the development of future bespoke and motivating rehabilitation apps.

## Methods

This study included shoulder pain–related apps (free and paid apps) that are available on the official stores for Apple (App Store) and Android (Google Play Store). These are the two major app stores that are currently available, accounting for a large sample of the top grossing apps [21]. The search was carried out in English.

The disease of interest was defined by using the following generic term: *shoulder pain*. The apps that focused on shoulder pain specifically were included in this study. Those that were related to another condition or had technical issues were excluded.

A total of 5 reviewers evaluated these apps by using the MARS. These five reviewers (JMRA, DPK, CEH, CN, EM) were chosen to avoid the potential subjectivity of a single reviewer. Two of these reviewers (CN and EM) had no previous medical background. As such, they were able to provide a layperson’s perspective on the information being provided in the apps. All apps were reviewed by each author during a consensus meeting to limit the introduction of heterogeneity in the decision-making process. This also allowed each app to be reviewed 5 times before a final decision was reached. The web-based platform Microsoft Forms was used to help complete the MARS. The MARS consists of 23 items, and each item is grouped into different sections related to apps—“Engagement,” “Functionality,” “Aesthetics,” “Information Quality,” and “Subjective Quality.” The MARS also contains an initial section for collecting general information on apps. There are 6 final items that can be adapted to specifically include information related to the topic of interest. Each item is scored from 1 (inadequate) to 5 (excellent). A final “Subjective Quality” section allows reviewers to give their personal opinions and recommendations for each app. This is used to give a measurement of overall app quality [22].

A descriptive analysis of the MARS scores was performed to provide an overview of the general quality of the available apps. Information for comparing the quality of content in free apps to that in paid apps was provided in the MARS. The layout of the MARS can be seen in Textbox 1.

**Mobile App Rating Scale structure.**

**Sections and definitions**

- Section A: *Engagement*
  App is fun, interesting, customizable, and interactive and is targeted to audience
- Section B: *Functionality*
  App functioning, app is easy to learn, navigation, flow logic, and gestural design
- Section C: *Aesthetics*
  Graphic design, visual appeal, color scheme, and style consistency
- Section D: *Information*
  Contains high-quality information from a credible source
- Section E: *App subjective quality*
  Personal interest in the app
- Section F: *App specific*
  Perceived impact of the app on the knowledge, attitudes, and intentions to change of the users, as well as the likelihood of actual change in the target health behavior

## Results

### Overview of Apps

Initially, 27 apps were chosen to be included in the final analysis. In the time between the initial search and the evaluation of the apps via the MARS, 2 apps were no longer available for access on the Google Play Store and were excluded from the final analysis. A total of 25 apps were included in the final analysis (8 from the App Store and 17 from the Google Play Store).

An overview of the main characteristics of each app included in this study is shown in Table 1. A star rating scale ranging from 1 to 5 was used, and the consensus among the reviewers resulted in an average star rating of 1.97, with no preferences for paid or free apps. The affiliations of most of the apps were commercial (22/25, 88%), except for one that was endorsed by a legitimate health care professional (Frozen Shoulder Protocols by Dr.Isaac’s Holistic Wellness). The apps mainly focused on physical exercises, with some providing further information related to shoulder pain conditions. Overall, there was no difference in app quality between the App Store and Google Play Store, suggesting that there were no preferences for one platform among the app developers. The results of the MARS can be seen in the following descriptive analysis sections.

**Table 1:** Mobile app characteristics.

App name	Platform	Developer	Price^a^
Clinical Pattern Recognition: Shoulder Pain	Android	PhysioU	£16.99
Frozen Shoulder Exercises	Android	abayapps	£0
Frozen Shoulder Exercises	Android	FeedTheGraph	£0
Frozen Shoulder Protocols	Android	Dr.Isaac’s Holistic Wellness	£0
Healure: Physiotherapy Exercise Plans	Android	Healure Technology	£0
Home Remedies for Shoulder Pain	Android	Ocean Digital Store	£0
Home Remedies for Shoulder Pain	Android	mikeg3590	£0
Neck & Shoulder Pain relief exercises, stretches	Android	OHealthApps Studio	£0
Neck Pain Exercises	Android	Mistertree Apps	£0
Neck, Shoulder Pain Relief	Android	HindiTreading Apps	£0
Rid of Shoulder Pain Remedies	Android	StatesApps	£0
Shoulder Pain	Android	Ciro Store	£0
Shoulder Pain Exercises	Android	adminapps	£0
Shoulder Pain Exercises	Android	tbeapps	£0
Shoulder Rehabilitation Exercises	Android	Sharudin	£0
Shoulder, Neck Pain Relief: Stretching Exercises	Android	Fitness Lab	£0
Shoulder Therapeutic Exercises	Android	Rixer	£0
Exercise Shoulder Pain	Apple	Medical	£0
MoovBuddy: Back, Neck & Posture	Apple	Digitallence	£0
NHS 24 MSK Help	Apple	NHS 24	£0
Shoulder Exercises for Seniors	Apple	Fitness for Seniors	£0
Physio in a Box	Apple	Medical	£0
Exercises for Shoulder Pain	Apple	Stefan Roobol	£1.99
Recognise Shoulder	Apple	Medical	£5.99
Healthy Shoulder 101	Apple	Xin Tan	£2.99

^a^A currency exchange rate of £1=US $1.23 is applicable.

### Engagement

**Figure 1:**
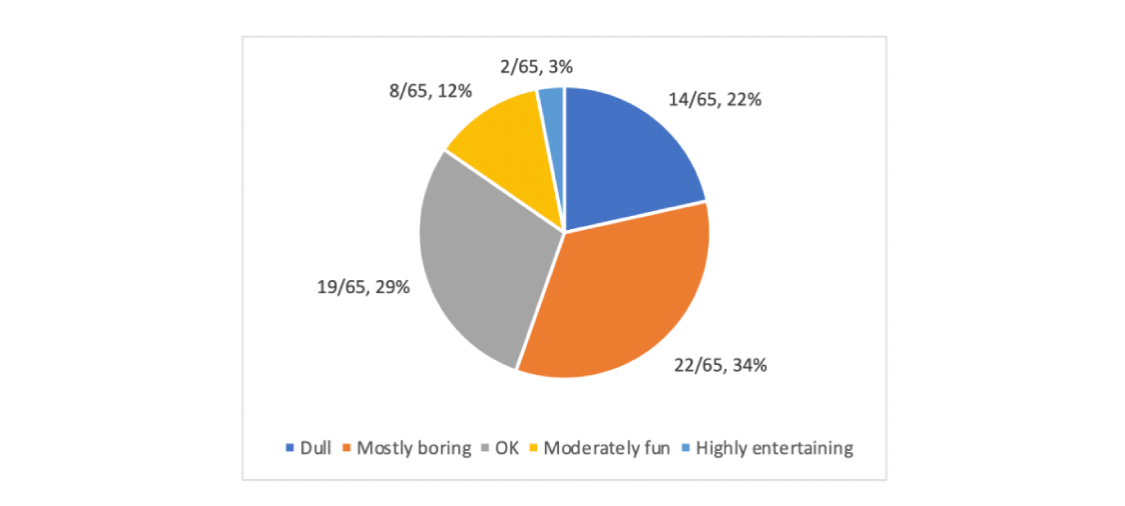
App entertainment.

Overall, the reviewers concluded that most apps lacked a lot of engaging features (21/25, 83%; Figure 1, Multimedia Appendices 1-4). In terms of the apps being fun to use, 22% (14/65) of apps were dull to use and not fun at all. Further, 34% (22/65) of the apps were boring and only slightly better, while 29% (19/65) were rated as being okay at best. With regard to the interest in the apps’ information presentation, 26% (17/65) of the apps were rated as not at all interesting, and 32% (21/65) were rated as mostly uninteresting. Additionally, 26% (17/65) were rated as okay.

With regard to the customization of the apps, a large majority of the apps (40/65, 62%) had no customization features; 15% (10/65) had very little features; and 17% (11/65) had very basic features, including a basic option for setting a list of exercises without a reminder option. Closely related to this is that 58% (38/65) of the apps were reported as having no interactivity features; 25% (16/65) had very few interactivity features; and 14% (9/65) had basic interactivity features, such as a calendar feature.

The final aspect of determining if an app was engaging was its appropriateness for its target audience. The majority of apps (34/65, 52%) were deemed to be acceptable but were not specific enough that they may be difficult for a layperson to engage with, and 22% (14/65) were reported as being acceptable enough that someone may be able to continue using the app without assistance. Further, 6% (4/65) were rated as not being appropriate at all, and 9% (6/65) were deemed to be perfectly suited to its intended audience.

### Functionality

**Figure 2:**
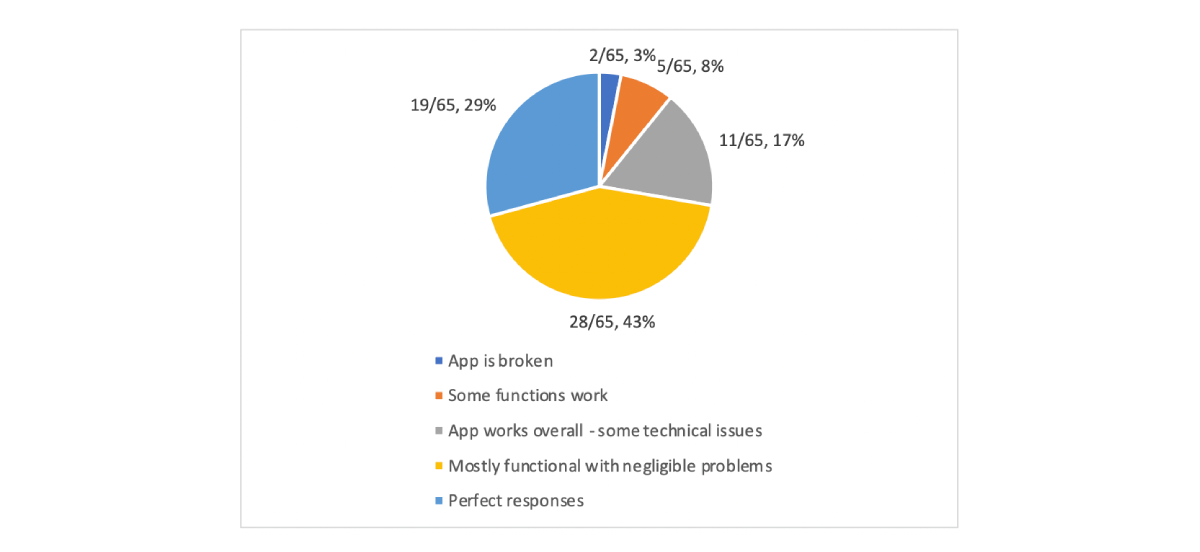
App performance.

The reviewers rated the same apps much higher in this section, as they were generally well built and easy to use (Figure 2, Multimedia Appendices 5-7). Overall, the performance of the apps was rated very highly, with 29% (19/65) of the apps having perfect performance and 43% (28/65) being rated as very good, with only minor issues such as slow loading speeds. Most of the apps were rated as being very easy to use with minimal guidance (27/65, 42%). Additionally, 26% (17/65) of the apps were rated perfectly in this section, as they could be used immediately with no effort, while 25% (16/65) required some time and effort.

Closely linked with an app’s ease of use is the navigation through each section of an app; 54% (35/65) of the apps were rated as easy to understand, with 17% (11/65) being perfect in terms of navigation, and 22% (14/65) were rated as good, only requiring minimal time to understand. The gestural design of the apps, such as the ability to zoom into pictures via pinching, was another functional aspect that most of the apps contained, with 23% (15/65) being rated as perfect. The majority (30/65, 46%) were mostly good, and 29% (19/65) were just okay, generally lacking the ability to zoom via pinching.

### Aesthetics

**Figure 3:**
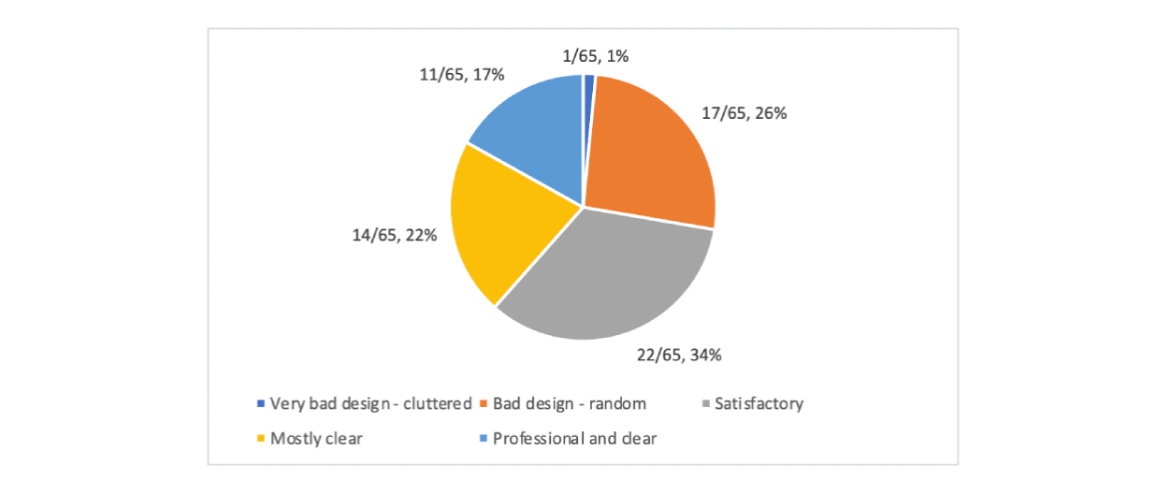
App layout.

The general aesthetics of the apps were rated as average (Figure 3, Multimedia Appendices 8 and 9). The layouts of the apps, such as the arrangement of the buttons and content on the screen, were rated as either satisfactory (22/65, 34%) or mostly clear (14/65, 22%). However, a large proportion (17/65, 26%) of the apps were rated as having a bad and unclear design that was difficult to navigate and understand. The quality of the graphics in the apps, which included pictures and videos, was mostly rated as moderate (26/65, 40%). The graphics in 20% (13/65) of the apps were high quality, and those in another 20% (13/65) were low quality, with pictures being either too small or too big to fit the screen correctly. The overall visual appeal was rated as average (26/65, 40%), with 25% (16/65) of the apps being very pleasant to look at and having an appropriate color scheme. However, 17% (11/65) were rated as ugly, and 15% (10/65) were rated as bad, having very poor designs such as oversized buttons and a loud, inappropriate color scheme.

### Information

**Figure 4:**
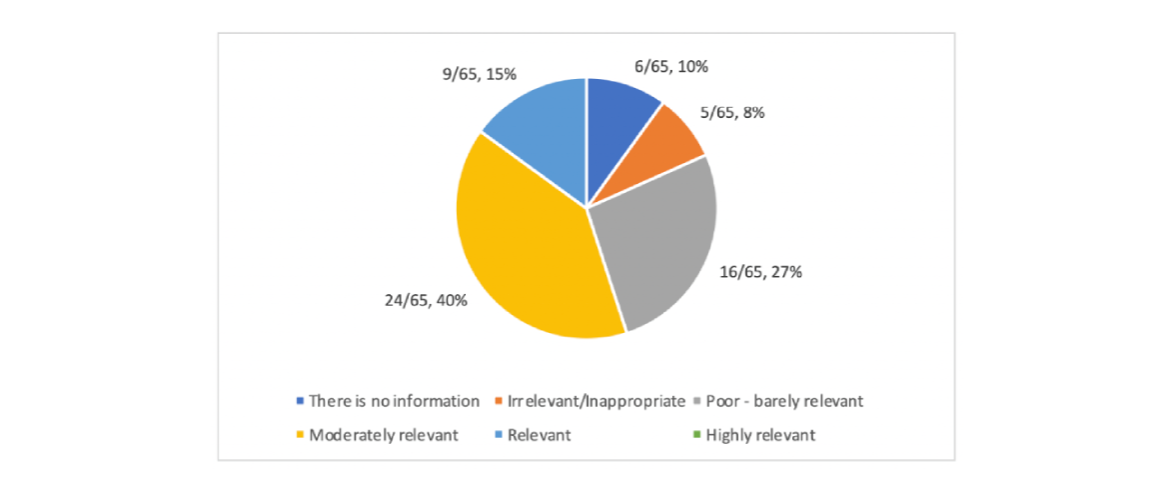
Quality of information.

The quality of the information provided by most of the apps (24/65, 37%) was rated as moderately relevant with potential for more concise information (Figure 4, Multimedia Appendices 10-12). Moreover, 25% (16/65) were rated as poor and inappropriate, with 9% (6/65) reported as having no available information. Among the apps that did provide information, the quantity was generally insufficient (21/65, 32%) or minimal (15/65, 23%). Additionally, 23% (15/65) of the apps provided a basic quantity of information, which was not comprehensive, and 6% (4/65) of the apps provided comprehensive and concise information. Further, 49% (32/65) of the apps provided a mostly clear and logical visual representation of information in the form pictures or videos, with 8% (5/65) providing perfectly clear graphs and pictures; 22% (14/65) provided visual information that was often unclear and not always logical; and 5% (3/65) provided no visual information at all.

Overall, the credibility of the sources of the information provided was rated as low, with the information in 29% (19/65) of the apps lacking any credibility at all, and the information in 6% (4/65) was believed to be from a suspicious source, as defined by the MARS scale. For 38% (25/65) of the apps, the legitimacy of their sources were questioned, but the apps were not believed to be suspicious, and 12% (8/65) provided no information regarding the credibility of their sources.

### Subjective Quality

**Figure 5:**
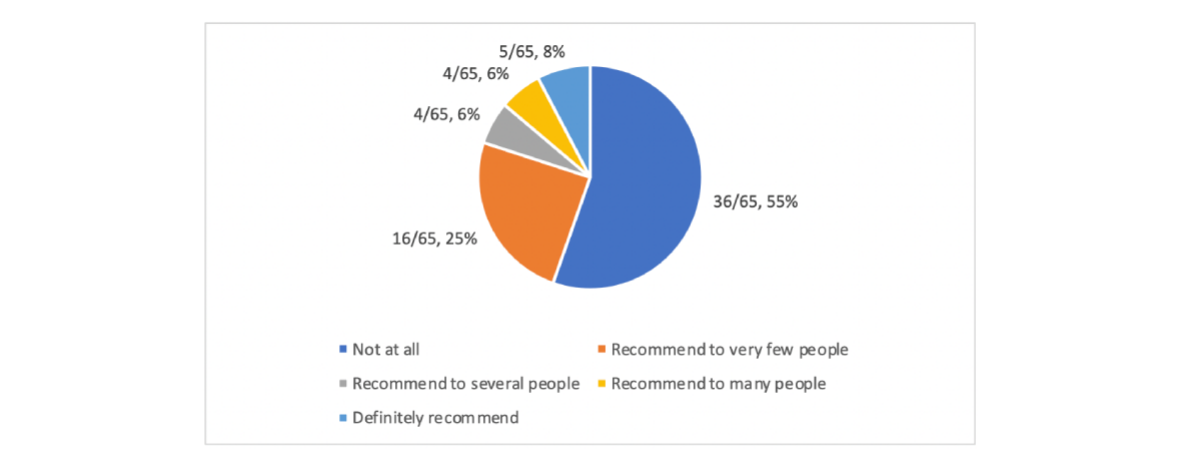
App recommendation.

This section provided information on the reviewers’ personal opinions on the apps being rated (Figure 5, Multimedia Appendices 13 and 14). When asked if they would recommend an app to someone who might benefit from it, the majority (36/65, 55%) responded with “not at all.” Additionally, 25% (16/65) of the apps would be recommended to very few people, and only 8% (5/65) of the apps would be definitely recommended.

This trend continued when the reviewers were asked how many times they would use the apps in the next 12 months if the apps were relevant to them; 66% (43/65) of responses were “none,” 9% (6/65) were “1-2” and “3-10,” and only 2% (1/65) were “more than 50 times.”

When the reviewers were asked if they would pay for an app, 89% (58/65) responded with “no,” with only 11% (7/65) responding “yes.”

## Discussion

### Principal Findings

This study presents a search and evaluation of smartphone apps related to shoulder pain that are available on the App Store and Google Play Store. An important point to note is that the mobile app market is very volatile and is constantly changing [23]. As this change is unpredictable, it is highly likely that the situation of the market at the time of the publication of this study will not be the same as the one presented herein. Throughout the duration of this study, changes in the market were detected. Principally, 2 apps were removed from this study, as they were no longer available on the Google Play Store and were unable to be evaluated by using the MARS. Despite this, the results presented in this study are the most accurate with regard to shoulder pain apps that were available at the time of writing and were evaluated using a validated assessment tool, such as the MARS.

Overall, the quality of the apps evaluated in this study was regarded as generally low, with the apps scoring an average star rating of 1.97 out of 5. The worst scores were related to what was offered to the users (“Engagement” and “Information” sections), and the best scores were related to the overall usability of the apps (“Functionality” and “Aesthetics” sections). This suggests that the apps are generally well built, with a few exceptions, but fail to interest the users. This statement is further reinforced with the apps’ subjective quality; over half of the reviewers would not recommend the apps (36/65, 55%) or use them again within the next 12 months (43/65, 66%). An overwhelming majority would also not pay for the apps (58/65, 89%), with some of the paid apps providing less information than their free counterparts and, in some instances, providing dangerous advice, such as recommending overhead exercises as the first stage of the rehabilitation of shoulder instability to a layperson with no previous medical knowledge, thereby putting them at risk of dislocation [24]. Within another paid app, links to videos caused the app to crash. In general, it was agreed among the reviewers that none of the apps would help to increase their knowledge or awareness of shoulder pain conditions or change their intentions to seek help and begin a rehabilitation program. Some of the apps contained too much information for the average user, and guidance would be advised before continuing their use. These apps also contained out-of-date references for their information, with some references being 30 years old.

The positive aspects of the apps that were outlined in the MARS included useful, working links to YouTube videos that provided further information on an appropriate exercise program for shoulder pain. Related to this is that some apps contained built-in video clips demonstrating the exercises being performed and provided a short written information section to allow the users to easily understand the exercises that they were being asked to perform. The general consensus was that a simple layout—one with clearly labeled buttons on the screen providing easier navigation throughout the app—was better. The most functional apps provided the option to sync the users’ exercises to their calendar, thereby providing a daily reminder notification to improve their adherence to an exercise program. The ability to increase the difficulty of the exercises was another useful functionality that was available in some of the apps to keep the users engaged for longer periods. The best apps generally had a larger range of exercises available, including body weight and weighted exercises for strength and stretching exercises.

### Limitations

This study has the usual limitations of these types of studies due to the nature of the products being studied (namely mobile apps). There is the possibility that some apps may have been missed that did not contain *shoulder pain* in their titles. Another limitation is the exclusion of the growing number of other mobile app stores outside of the main two, such as the Huawei App Store in China [25]. This in turn resulted in potentially relevant apps that are in a foreign language and are available in only specific regions being excluded from this study. The inclusion of paid apps, which may have access to additional customization options, could potentially lead to bias in favor of these apps when compared to their free counterparts. A reliance on the product summaries and subjective rating tools used in app stores leads to another risk of bias with regard to the quality of the product due to a lack of validation [26].

### Conclusion

The shoulder pain–related apps that are currently available are generally well built technically but fail to offer an appropriate quantity and quality of information to the users. Therefore, they fail to have an engaging impact. The vast majority of such apps are not based on scientific evidence, with the few exceptions being vastly outdated. They are unlikely to have been rigorously tested, putting into question the safety and confidentiality of the information being collected from users. There is also a low level of health care professional involvement in the development process, which could result in potential safety issues for users if the information provided by an app is not legitimate. Future apps that are being developed should aim to improve on these aspects while taking advantage of the constant innovation of mobile technology, such as integration with wearable devices to track activity levels and increase exercise adherence [27,28]. Simple measures, such as recognized quality assurance standards and external reviews, should also be proposed [29].

## Appendix

Multimedia Appendix 1 App interest.

Multimedia Appendix 2 App customization.

Multimedia Appendix 3 App interactivity.

Multimedia Appendix 4 Target group.

Multimedia Appendix 5 Ease of use.

Multimedia Appendix 6 App navigation.

Multimedia Appendix 7 Gestural design.

Multimedia Appendix 8 App graphics.

Multimedia Appendix 9 Visual appeal.

Multimedia Appendix 10 Quantity of information.

Multimedia Appendix 11 Visual information.

Multimedia Appendix 12 Credibility of source.

Multimedia Appendix 13 App usage over 12 months.

Multimedia Appendix 14 Would you pay for the app?

## Abbreviations

MARS: Mobile App Rating Scale
