# Supplementary material for: Rating the Quality of Smartphone Apps Related to Shoulder Pain: Systematic Search and Evaluation Using the Mobile App Rating Scale
Source: JMIR Form Res. 2022 May 26;6(5):e34339. doi: 10.2196/34339 (PMC9185331; doi:10.2196/34339)
Supplement: Supplementary file 1 [file formative_v6i5e34339_app1.docx]

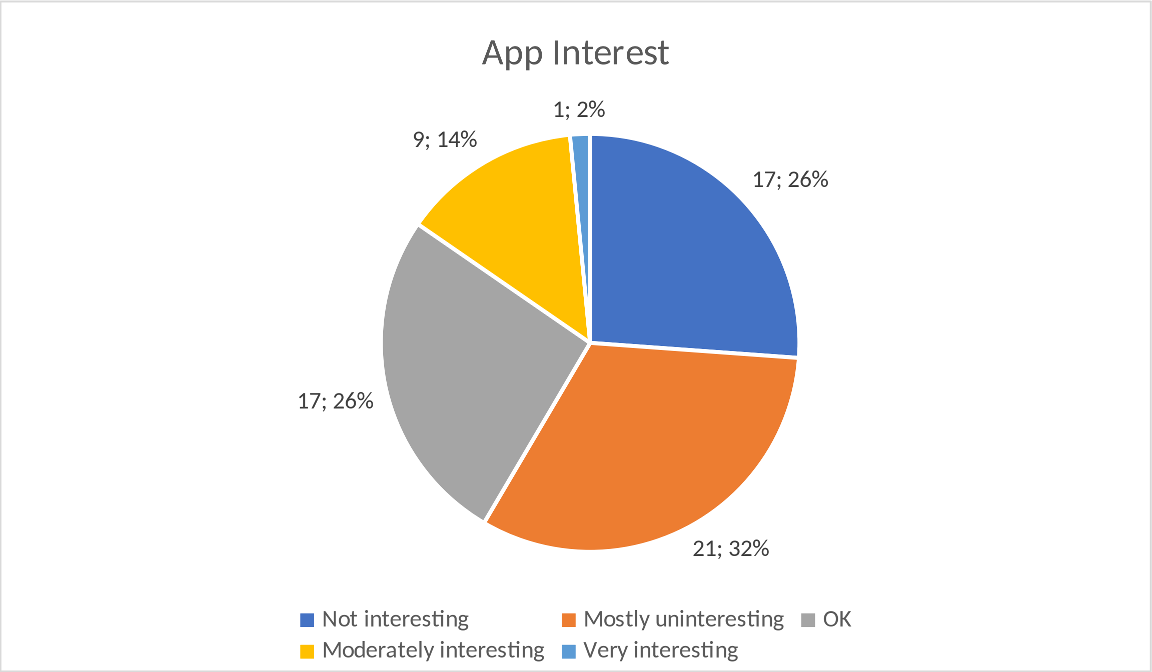
App Interest
1; 2%
9; 14%
17; 26%
17; 26%
21; 32%
Not interesting
Mostly uninteresting
OK
Moderately interesting
Very interesting
