# Supplementary material for: Rating the Quality of Smartphone Apps Related to Shoulder Pain: Systematic Search and Evaluation Using the Mobile App Rating Scale
Source: JMIR Form Res. 2022 May 26;6(5):e34339. doi: 10.2196/34339 (PMC9185331; doi:10.2196/34339)
Supplement: Supplementary file 2 [file formative_v6i5e34339_app10.docx]

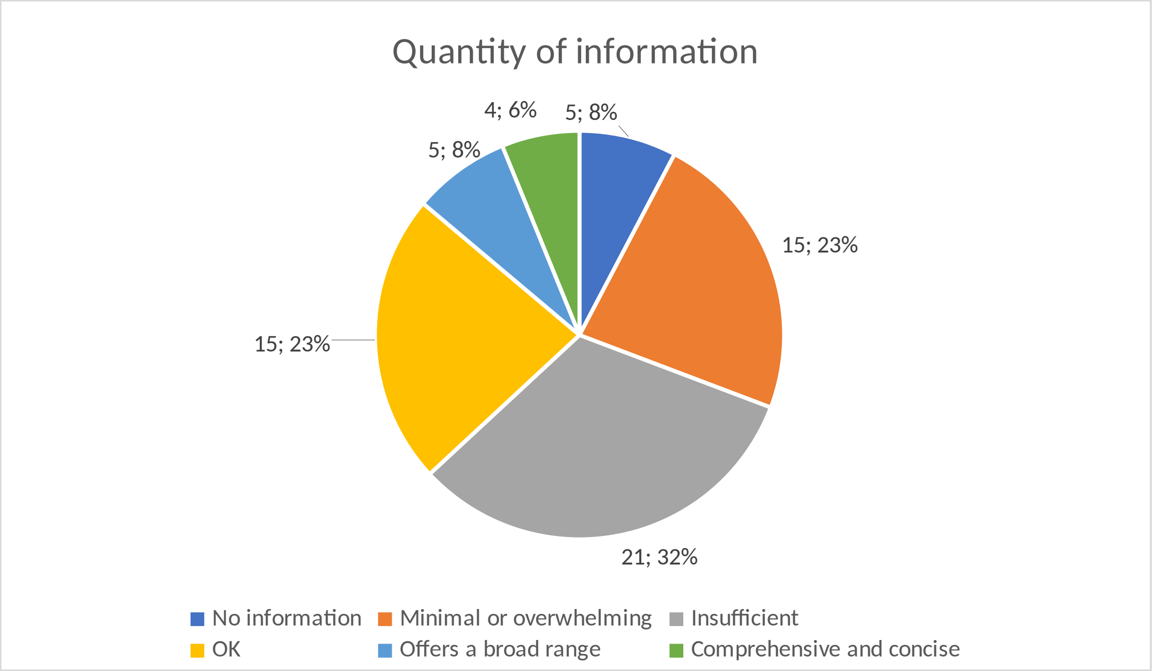
Quantity of information
5; 8%
15; 23%
21; 32%
15; 23%
5; 8%
4; 6%
No information
Minimal or overwhelming
Insufficient
OK
Offers a broad range
Comprehensive and concise
