# Supplementary material for: Rating the Quality of Smartphone Apps Related to Shoulder Pain: Systematic Search and Evaluation Using the Mobile App Rating Scale
Source: JMIR Form Res. 2022 May 26;6(5):e34339. doi: 10.2196/34339 (PMC9185331; doi:10.2196/34339)
Supplement: Supplementary file 3 [file formative_v6i5e34339_app11.docx]

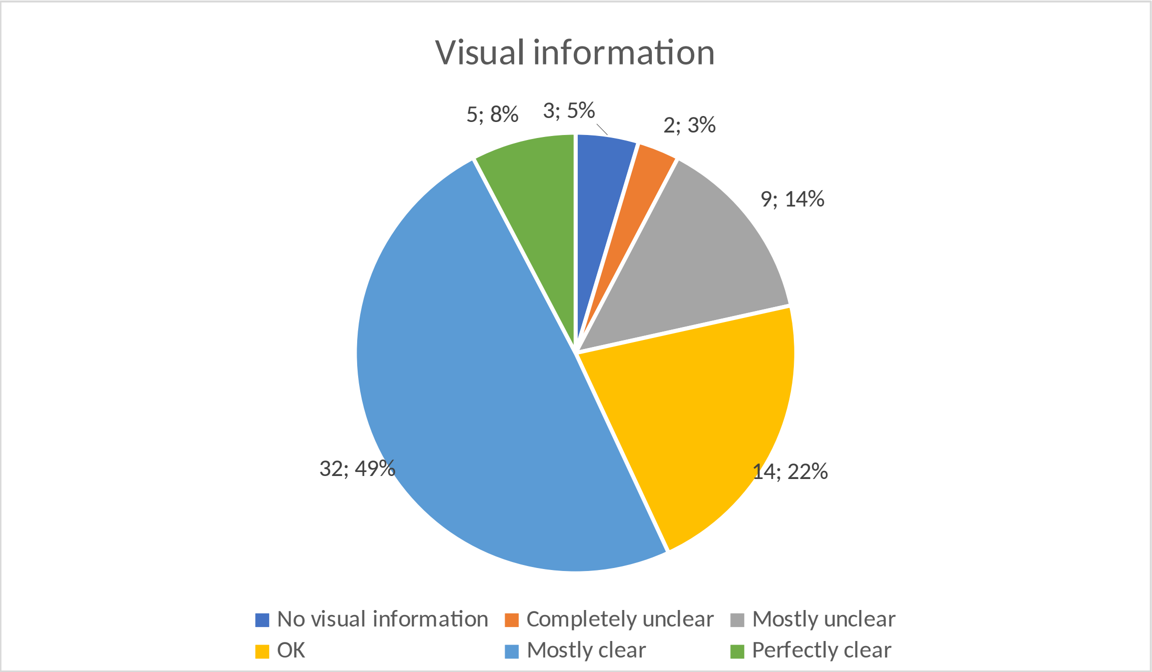

Visual information
5; 8%
3; 5%
2; 3%
9; 14%
32; 49%
14; 22%
No visual information
Completely unclear
Mostly unclear
OK
Mostly clear
Perfectly clear
