# Supplementary material for: Rating the Quality of Smartphone Apps Related to Shoulder Pain: Systematic Search and Evaluation Using the Mobile App Rating Scale
Source: JMIR Form Res. 2022 May 26;6(5):e34339. doi: 10.2196/34339 (PMC9185331; doi:10.2196/34339)
Supplement: Supplementary file 4 [file formative_v6i5e34339_app12.docx]

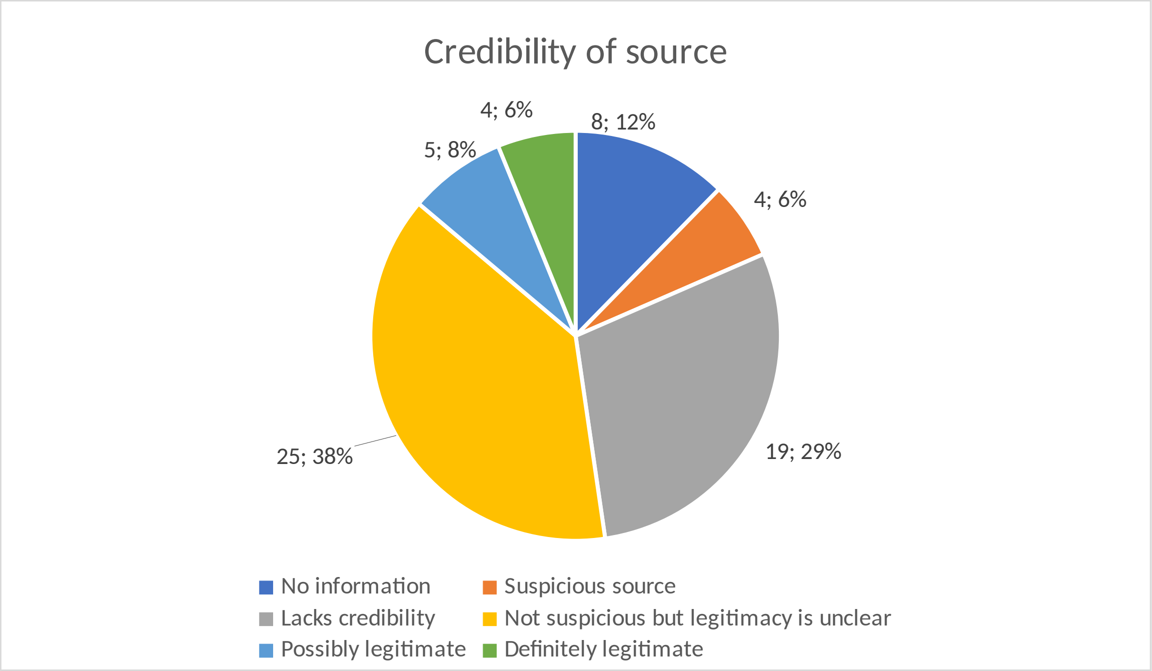
Credibility of source
8; 12%
4; 6%
19; 29%
25; 38%
5; 8%
4; 6%
No information
Suspicious source
Lacks credibility
Not suspicious but legitimacy is unclear
Possibly legitimate
Definitely legitimate
