# Supplementary material for: Rating the Quality of Smartphone Apps Related to Shoulder Pain: Systematic Search and Evaluation Using the Mobile App Rating Scale
Source: JMIR Form Res. 2022 May 26;6(5):e34339. doi: 10.2196/34339 (PMC9185331; doi:10.2196/34339)
Supplement: Supplementary file 5 [file formative_v6i5e34339_app13.docx]

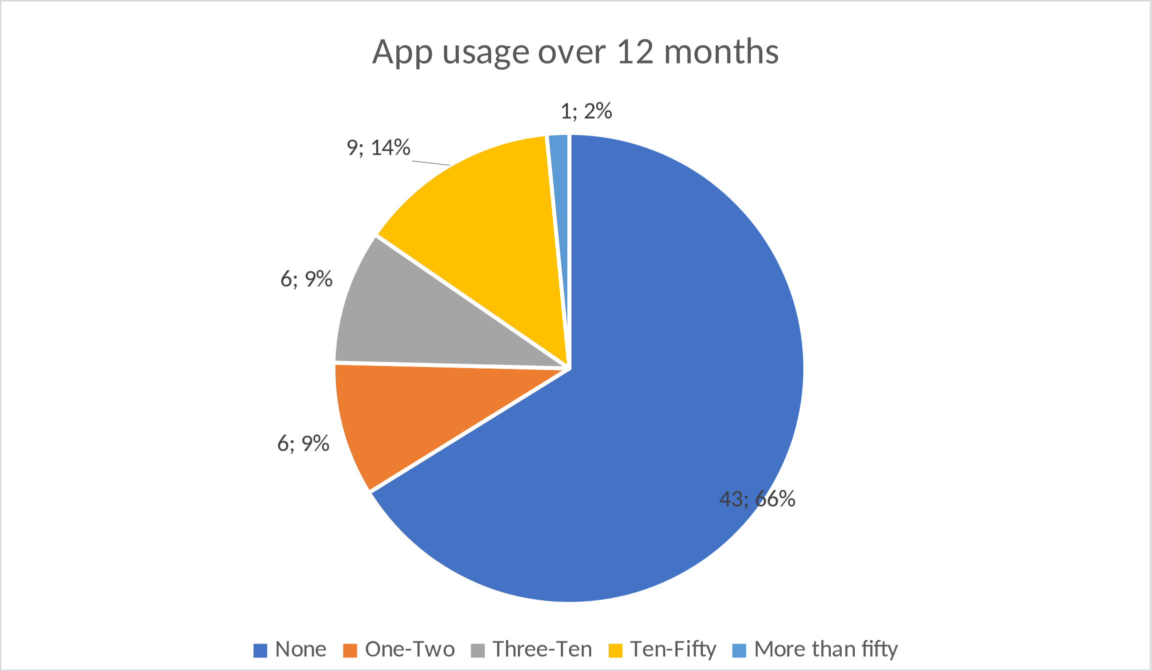
App usage over 12 months
1; 2%
9; 14%
6; 9%
6; 9%
43; 66%
None
One-Two
Three-Ten
Ten-Fifty
More than fifty
