# Supplementary material for: Rating the Quality of Smartphone Apps Related to Shoulder Pain: Systematic Search and Evaluation Using the Mobile App Rating Scale
Source: JMIR Form Res. 2022 May 26;6(5):e34339. doi: 10.2196/34339 (PMC9185331; doi:10.2196/34339)
Supplement: Supplementary file 6 [file formative_v6i5e34339_app14.docx]

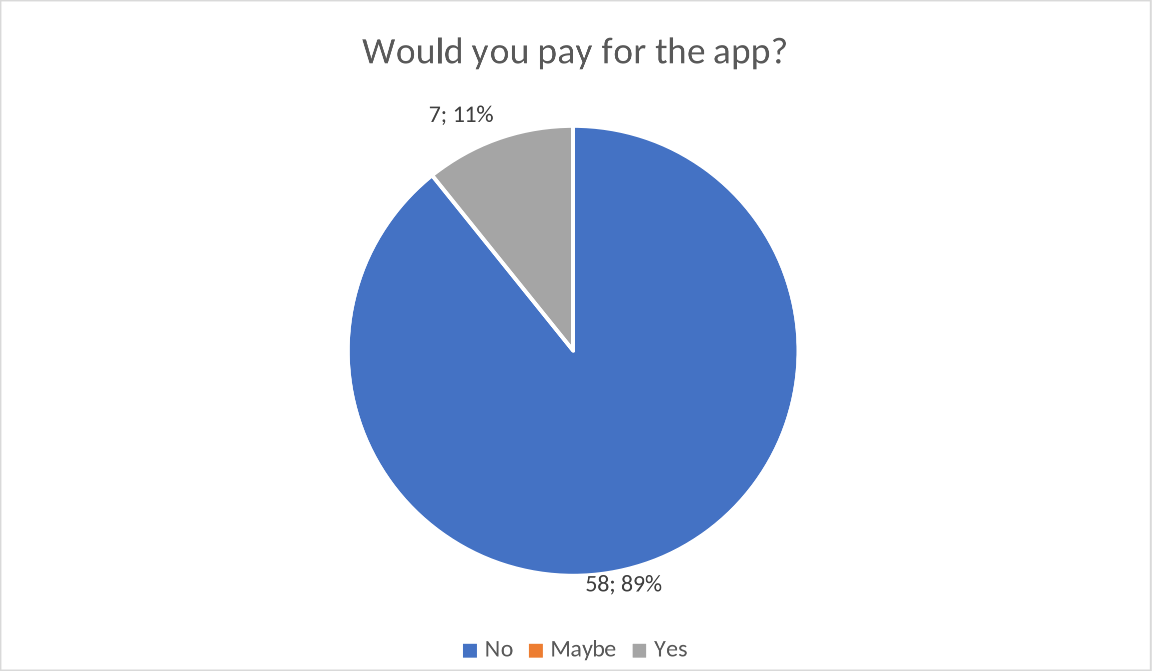
Would you pay for the app?
7; 11%
58; 89%
No
Maybe
Yes
