# Supplementary material for: Rating the Quality of Smartphone Apps Related to Shoulder Pain: Systematic Search and Evaluation Using the Mobile App Rating Scale
Source: JMIR Form Res. 2022 May 26;6(5):e34339. doi: 10.2196/34339 (PMC9185331; doi:10.2196/34339)
Supplement: Supplementary file 7 [file formative_v6i5e34339_app2.docx]

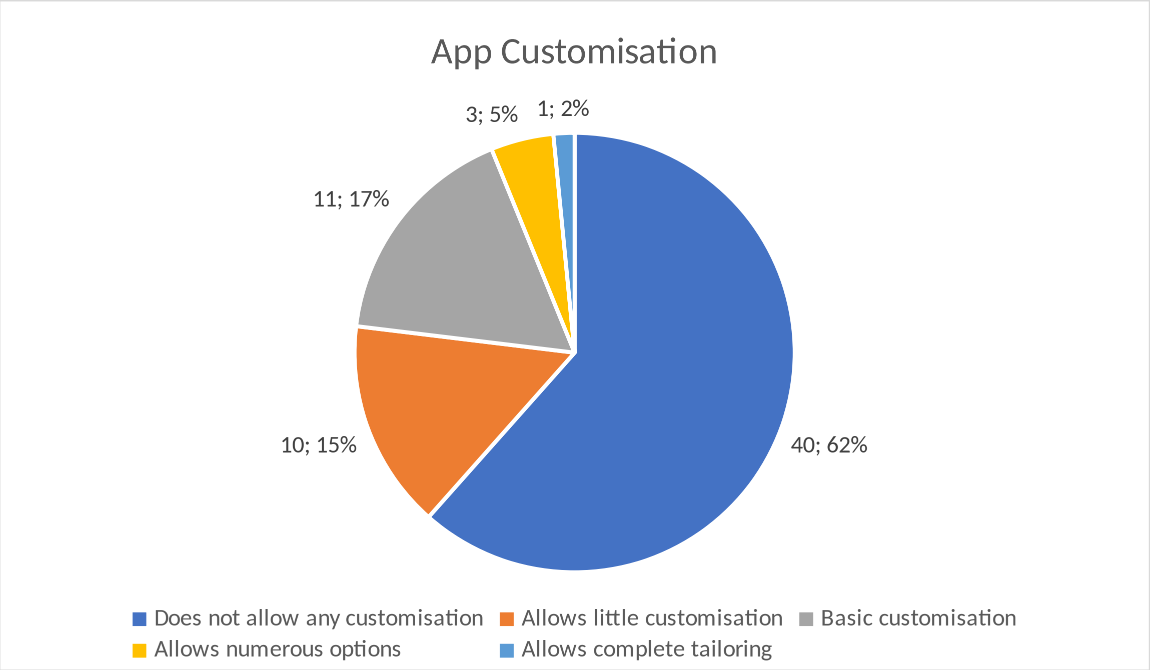
App Customisation
3; 5%
1; 2%
11; 17%
10; 15%
40; 62%
Does not allow any customisation
Allows little customisation
Basic customisation
Allows numerous options
Allows complete tailoring
