# Supplementary material for: Rating the Quality of Smartphone Apps Related to Shoulder Pain: Systematic Search and Evaluation Using the Mobile App Rating Scale
Source: JMIR Form Res. 2022 May 26;6(5):e34339. doi: 10.2196/34339 (PMC9185331; doi:10.2196/34339)
Supplement: Supplementary file 8 [file formative_v6i5e34339_app3.docx]

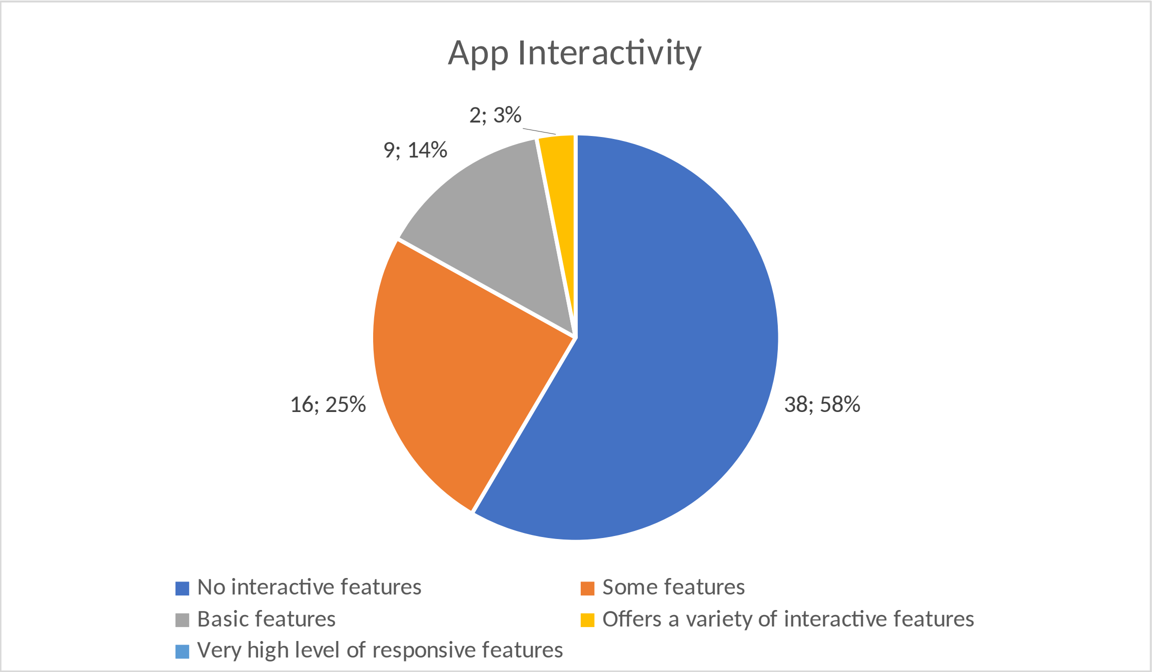
App Interactivity
2; 3%
9; 14%
16; 25%
38; 58%
No interactive features
Some features
Basic features
Offers a variety of interactive features
Very high level of responsive features
