# Supplementary material for: Rating the Quality of Smartphone Apps Related to Shoulder Pain: Systematic Search and Evaluation Using the Mobile App Rating Scale
Source: JMIR Form Res. 2022 May 26;6(5):e34339. doi: 10.2196/34339 (PMC9185331; doi:10.2196/34339)
Supplement: Supplementary file 9 [file formative_v6i5e34339_app4.docx]

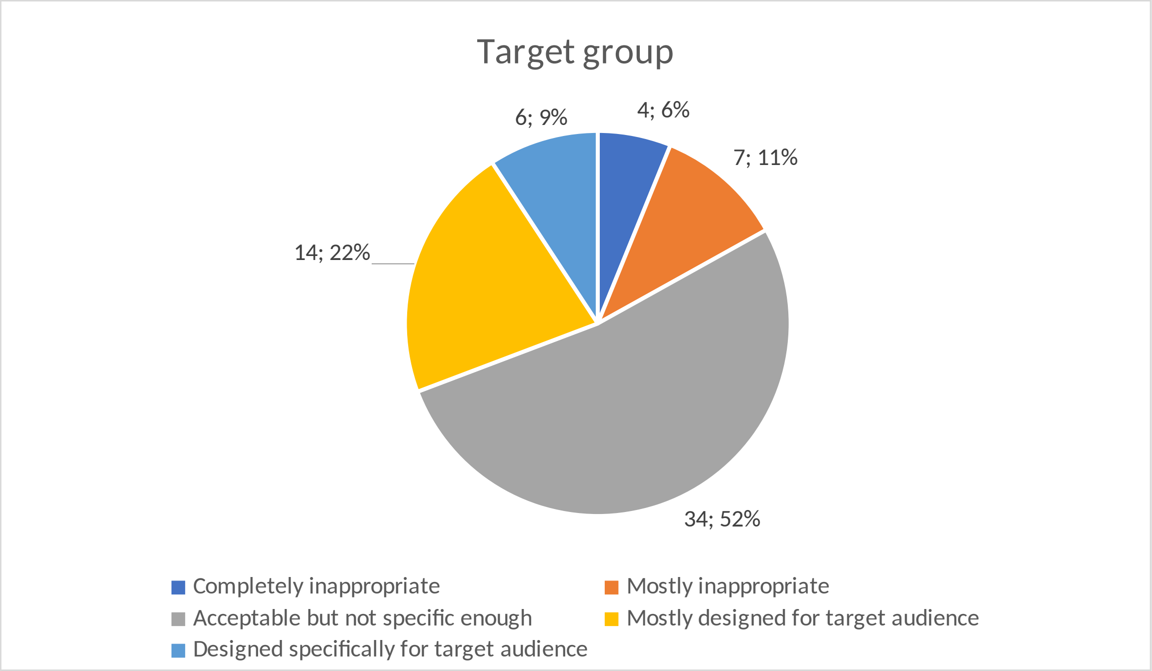
Target group
4; 6%
7; 11%
34; 52%
14; 22%
6; 9%
Completely inappropriate
Mostly inappropriate
Acceptable but not specific enough
Mostly designed for target audience
Designed specifically for target audience
