# Supplementary material for: Rating the Quality of Smartphone Apps Related to Shoulder Pain: Systematic Search and Evaluation Using the Mobile App Rating Scale
Source: JMIR Form Res. 2022 May 26;6(5):e34339. doi: 10.2196/34339 (PMC9185331; doi:10.2196/34339)
Supplement: Supplementary file 10 [file formative_v6i5e34339_app5.docx]

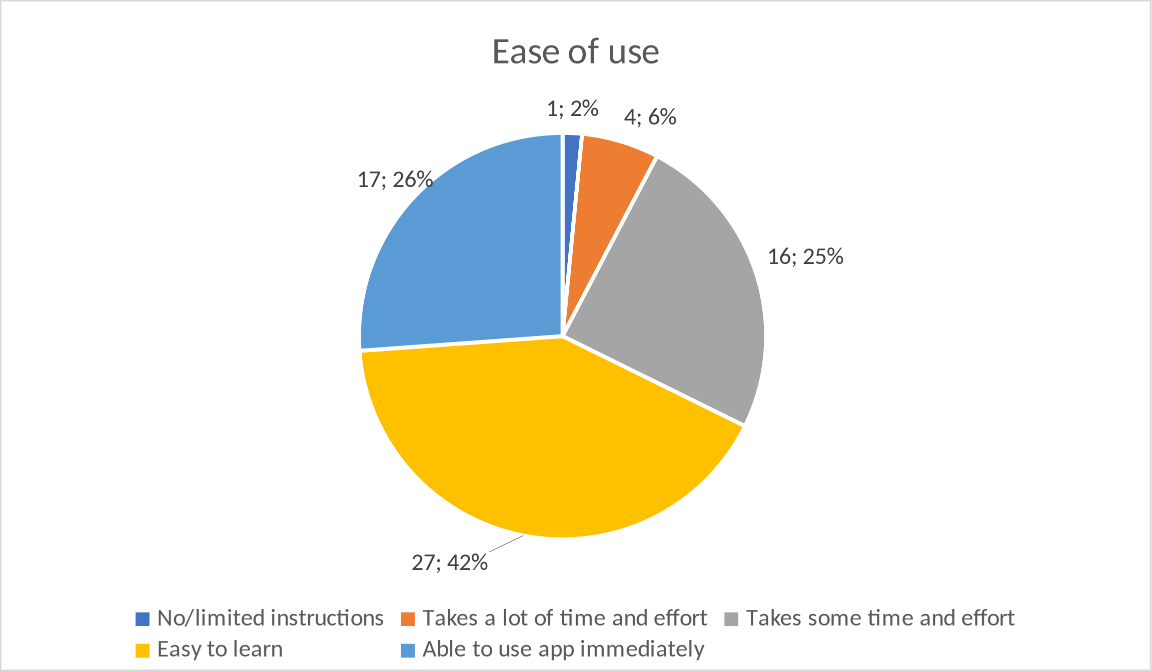

Ease of use
1; 2%
4; 6%
16; 25%
27; 42%
17; 26%
No/limited instructions
Takes a lot of time and effort
Takes some time and effort
Easy to learn
Able to use app immediately
