# Supplementary material for: Rating the Quality of Smartphone Apps Related to Shoulder Pain: Systematic Search and Evaluation Using the Mobile App Rating Scale
Source: JMIR Form Res. 2022 May 26;6(5):e34339. doi: 10.2196/34339 (PMC9185331; doi:10.2196/34339)
Supplement: Supplementary file 11 [file formative_v6i5e34339_app6.docx]

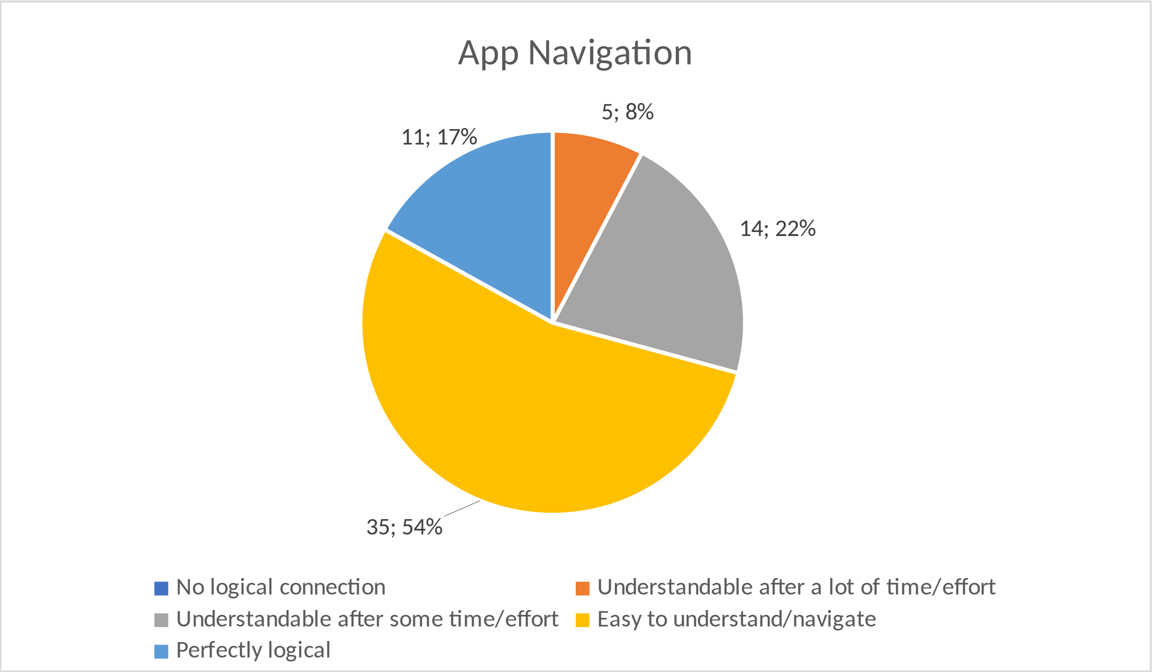
App Navigation
5; 8%
14; 22%
11; 17%
35; 54%
No logical connection
Understandable after a lot of time/effort
Understandable after some time/effort
Easy to understand/navigate
Perfectly logical
