# Supplementary material for: Rating the Quality of Smartphone Apps Related to Shoulder Pain: Systematic Search and Evaluation Using the Mobile App Rating Scale
Source: JMIR Form Res. 2022 May 26;6(5):e34339. doi: 10.2196/34339 (PMC9185331; doi:10.2196/34339)
Supplement: Supplementary file 12 [file formative_v6i5e34339_app7.docx]

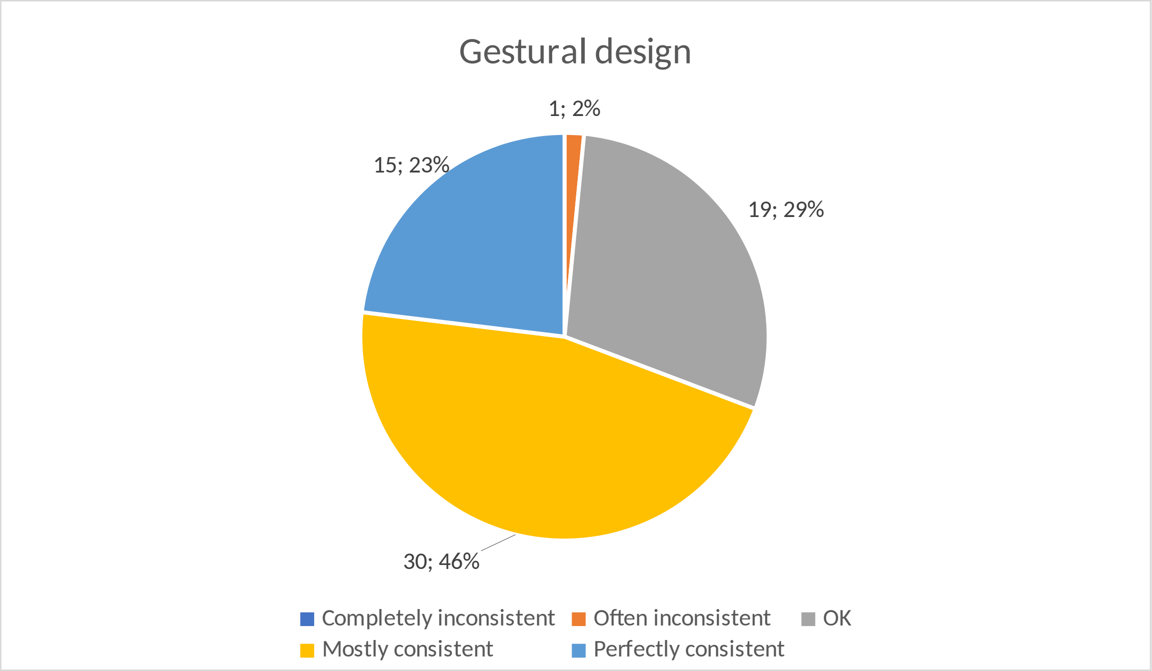
Gestural design
1; 2%
19; 29%
30; 46%
15; 23%
Completely inconsistent
Often inconsistent
OK
Mostly consistent
Perfectly consistent
