# Supplementary material for: Rating the Quality of Smartphone Apps Related to Shoulder Pain: Systematic Search and Evaluation Using the Mobile App Rating Scale
Source: JMIR Form Res. 2022 May 26;6(5):e34339. doi: 10.2196/34339 (PMC9185331; doi:10.2196/34339)
Supplement: Supplementary file 13 [file formative_v6i5e34339_app8.docx]

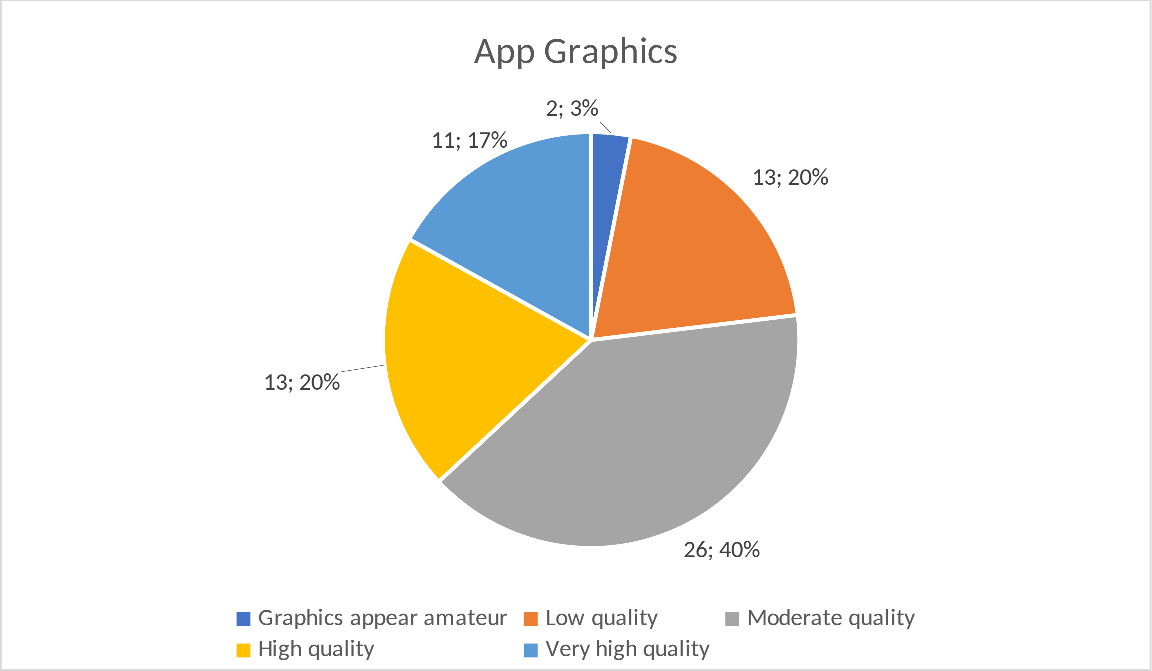
App Graphics
2; 3%
13; 20%
26; 40%
13; 20%
11; 17%
Graphics appear amateur
Low quality
Moderate quality
High quality
Very high quality
