# Supplementary material for: Rating the Quality of Smartphone Apps Related to Shoulder Pain: Systematic Search and Evaluation Using the Mobile App Rating Scale
Source: JMIR Form Res. 2022 May 26;6(5):e34339. doi: 10.2196/34339 (PMC9185331; doi:10.2196/34339)
Supplement: Supplementary file 14 [file formative_v6i5e34339_app9.docx]

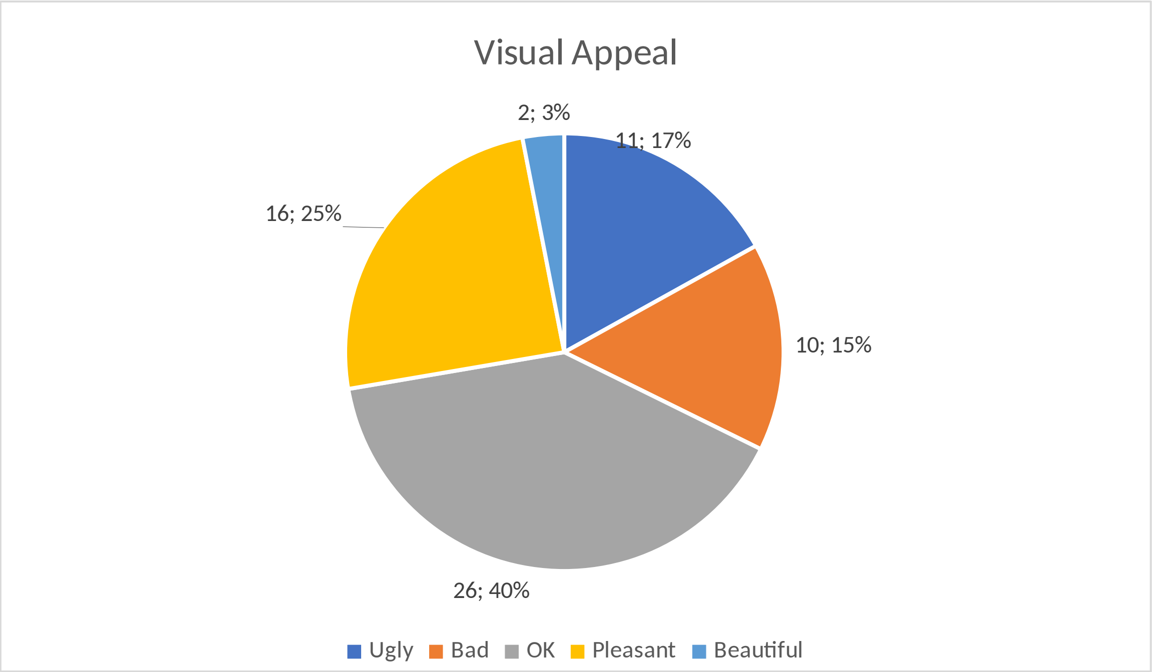

Visual Appeal
2; 3%
11; 17%
16; 25%
10; 15%
26; 40%
Ugly
Bad
OK
Pleasant
Beautiful
